# Supplementary material for: Transcriptomics and Metabolomics of Reactive Oxygen Species Modulation in Near-Null Magnetic Field-Induced Arabidopsis thaliana
Source: Biomolecules. 2022 Dec 6;12(12):1824. doi: 10.3390/biom12121824 (PMC9775259; doi:10.3390/biom12121824)
Supplement: Supplementary file 1 [file biomolecules-12-01824-s001.zip › Supplementary Dataset S1.pdf]

Supplementary Dataset S1

Gene expression changes, statistical analysis and fold change volcano plots for all time points assessed

10 min

Test Description

Selected Test : T Test Against Zero
p-value computation: Asymptotic
Multiple Testing Correction: Benjamini-Hochberg

Result Summary

	P all	P < 0.05	P < 0.02	P < 0.01	P < 0.0050	P < 0.0010
FC all	37538	10811	9153	8207	7362	5769
FC > 1.1	18950	10307	8932	8093	7297	5753
FC > 1.5	9827	8982	8181	7616	6974	5636
FC > 2.0	3654	3591	3499	3392	3219	2809
FC > 3.0	142	136	120	93	52	21
Expected by ch...		540	183	82	36	5

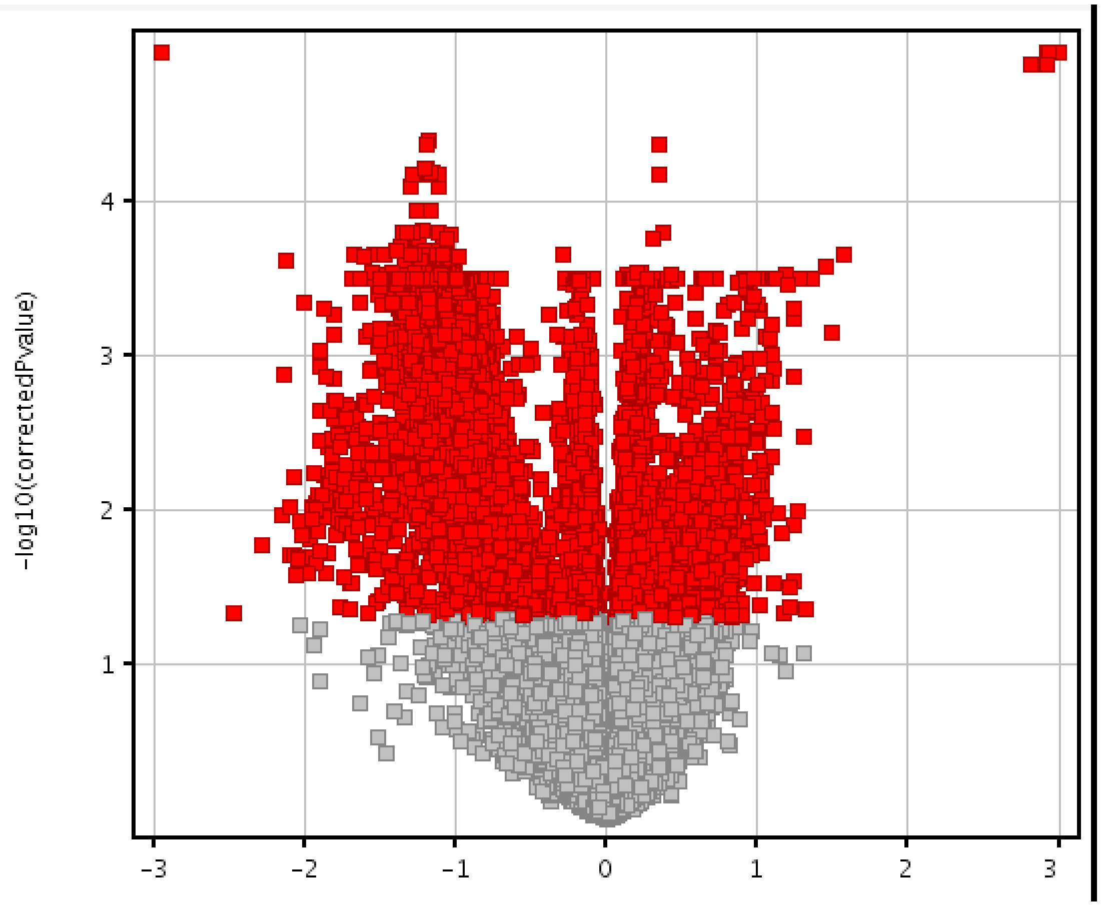

1 h

Test Description

Selected Test : T Test Against Zero
p-value computation: Asymptotic
Multiple Testing Correction: Benjamini-Hochberg

Result Summary

	P all	P < 0.05	P < 0.02	P < 0.01	P < 0.0050	P < 0.0...
FC all	37538	10321	8725	7887	7212	5997
FC > 1.1	20051	9900	8570	7810	7176	5991
FC > 1.5	10063	8593	7915	7423	6960	5939
FC > 2.0	6713	6653	6551	6431	6294	5698
FC > 3.0	29	25	24	21	19	15
Expected ...		516	174	78	36	5

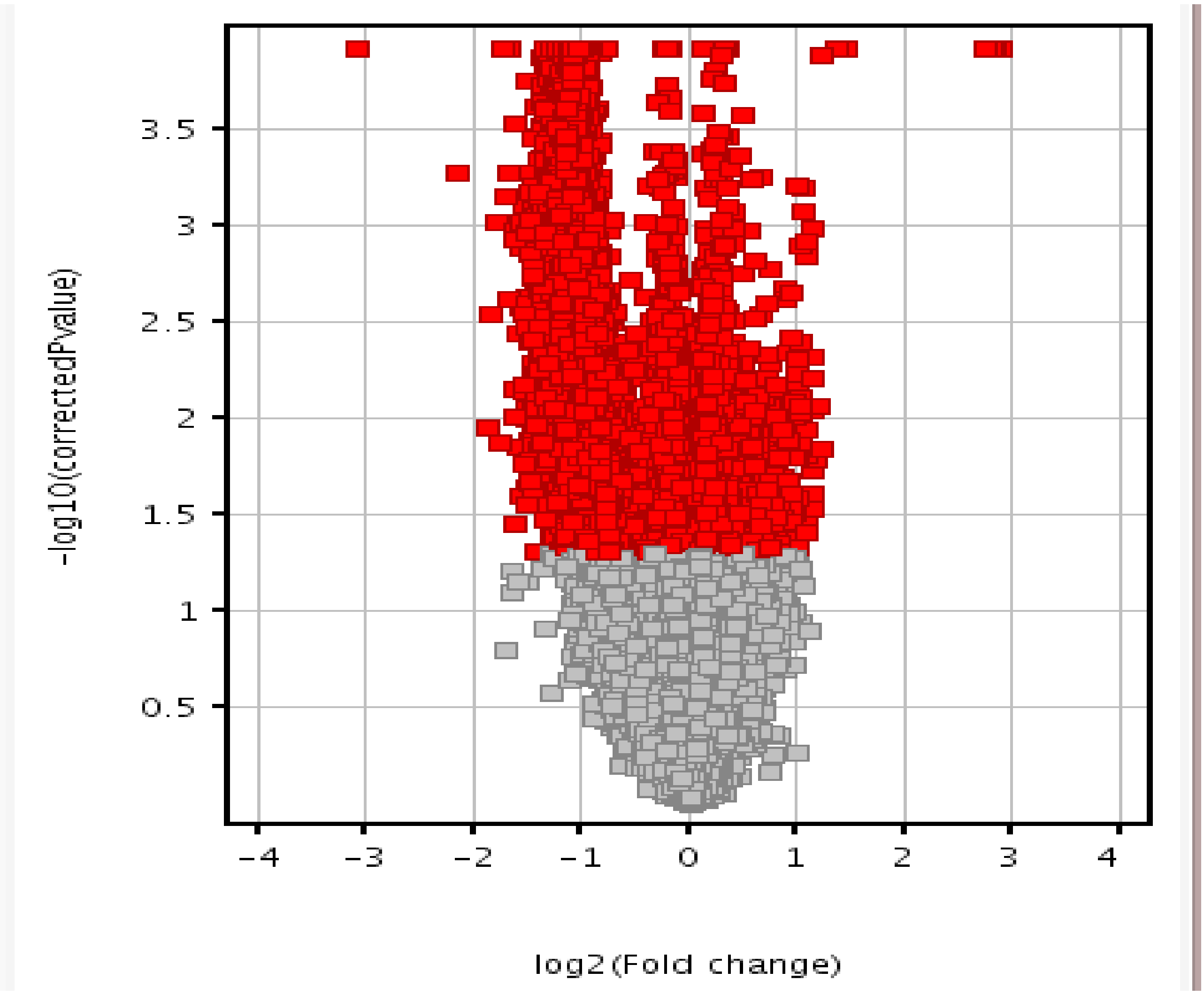

2 h

Test Description

Selected Test : T Test Against Zero
p-value computation: Asymptotic
Multiple Testing Correction: Benjamini-Hochberg

Result Summary

	P all	P < 0.05	P < 0.02	P < 0.01	P < 0.0050	P < 0.0010
FC all	37538	10876	9084	8123	7363	6133
FC > 1.1	18830	10118	8760	7950	7288	6119
FC > 1.5	9748	8521	7812	7319	6883	5976
FC > 2.0	6286	6248	6174	6086	5974	5612
FC > 3.0	21	19	18	15	15	13
Expected by ch...		543	181	81	36	6

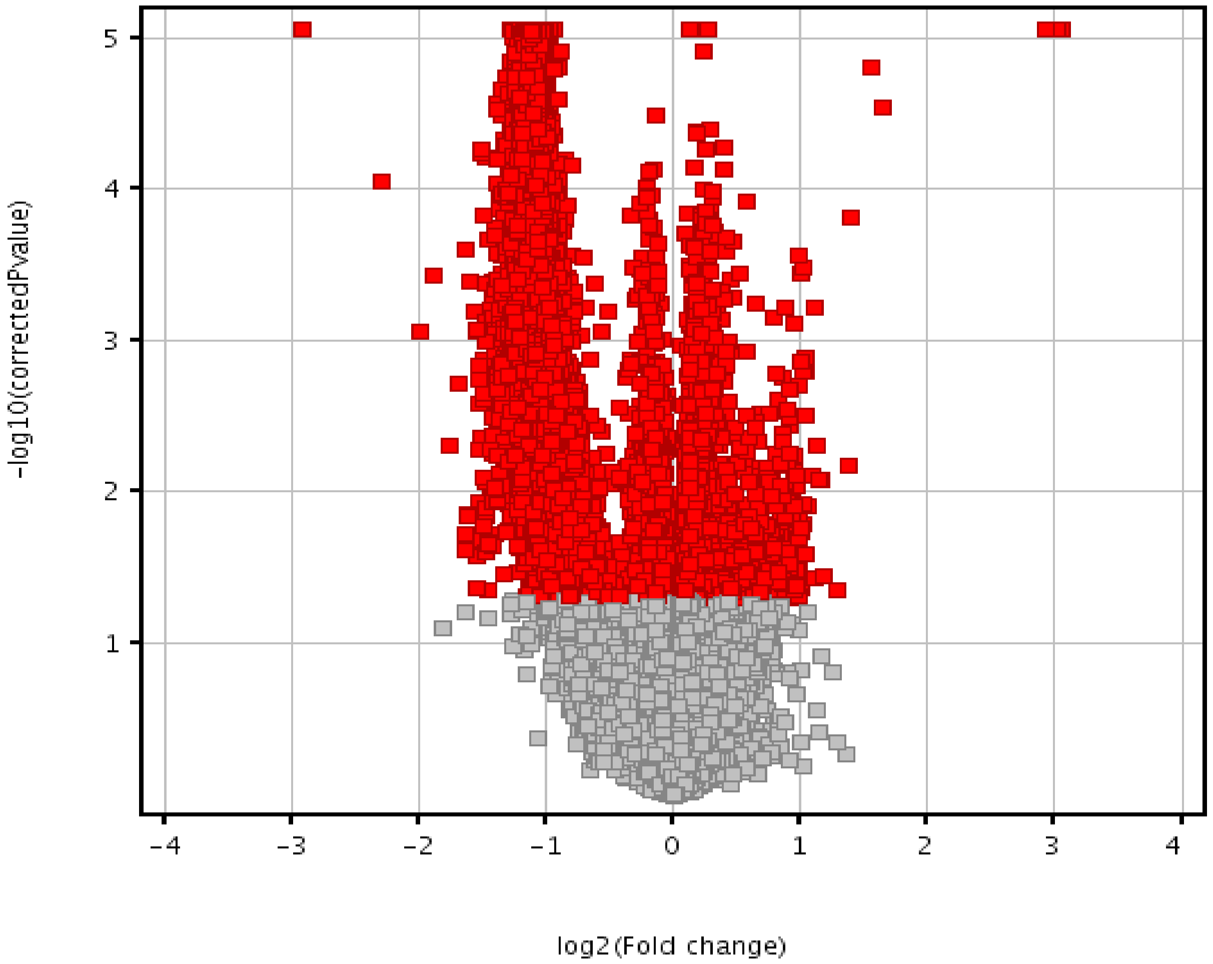

4 h

Test Description

Selected Test : T Test Against Zero
p-value computation: Asymptotic
Multiple Testing Correction: Benjamini-Hochberg

Result Summary

	P all	P < 0.05	P < 0.02	P < 0.01	P < 0.0050	P < 0.0010
FC all	37538	11135	9057	7982	7136	5759
FC > 1.1	21896	10709	8901	7908	7098	5753
FC > 1.5	9557	8557	7826	7298	6770	5666
FC > 2.0	2056	2018	1965	1904	1804	1464
FC > 3.0	21	16	14	14	13	11
Expected by ch...		556	181	79	35	5

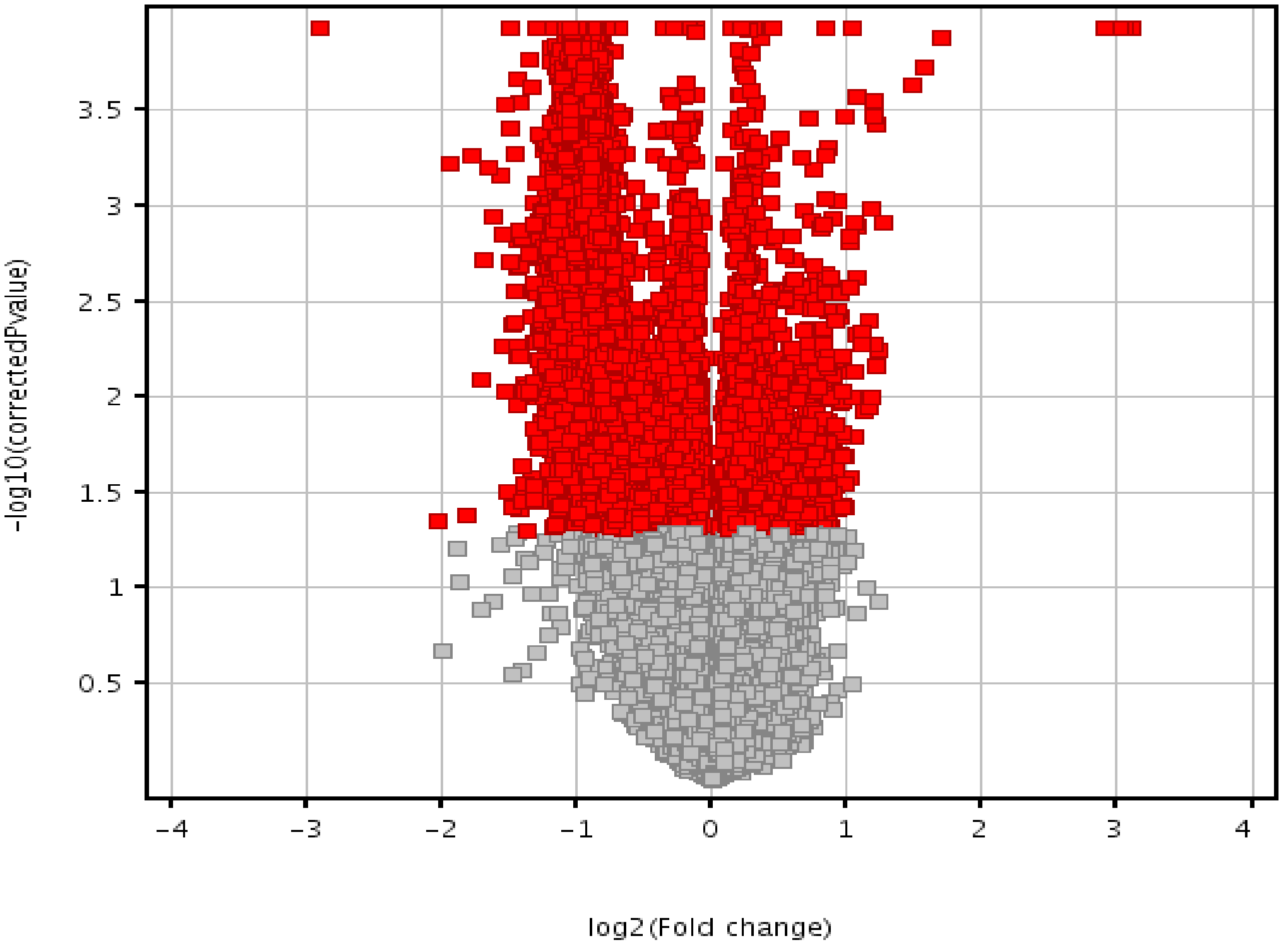

24 h

Test Description

Selected Test : T Test Against Zero
p-value computation: Asymptotic
Multiple Testing Correction: Benjamini-Hochberg

Result Summary

	P all	P < 0.05	P < 0.02	P < 0.01	P < 0.0050	P < 0.0010
FC all	37538	7984	6861	6171	5597	0
FC > 1.1	19702	7900	6836	6163	5590	0
FC > 1.5	9544	7443	6648	6065	5539	0
FC > 2.0	4244	4104	3972	3849	3712	0
FC > 3.0	12	11	10	9	9	0
Expected by ch...		399	137	61	27	0

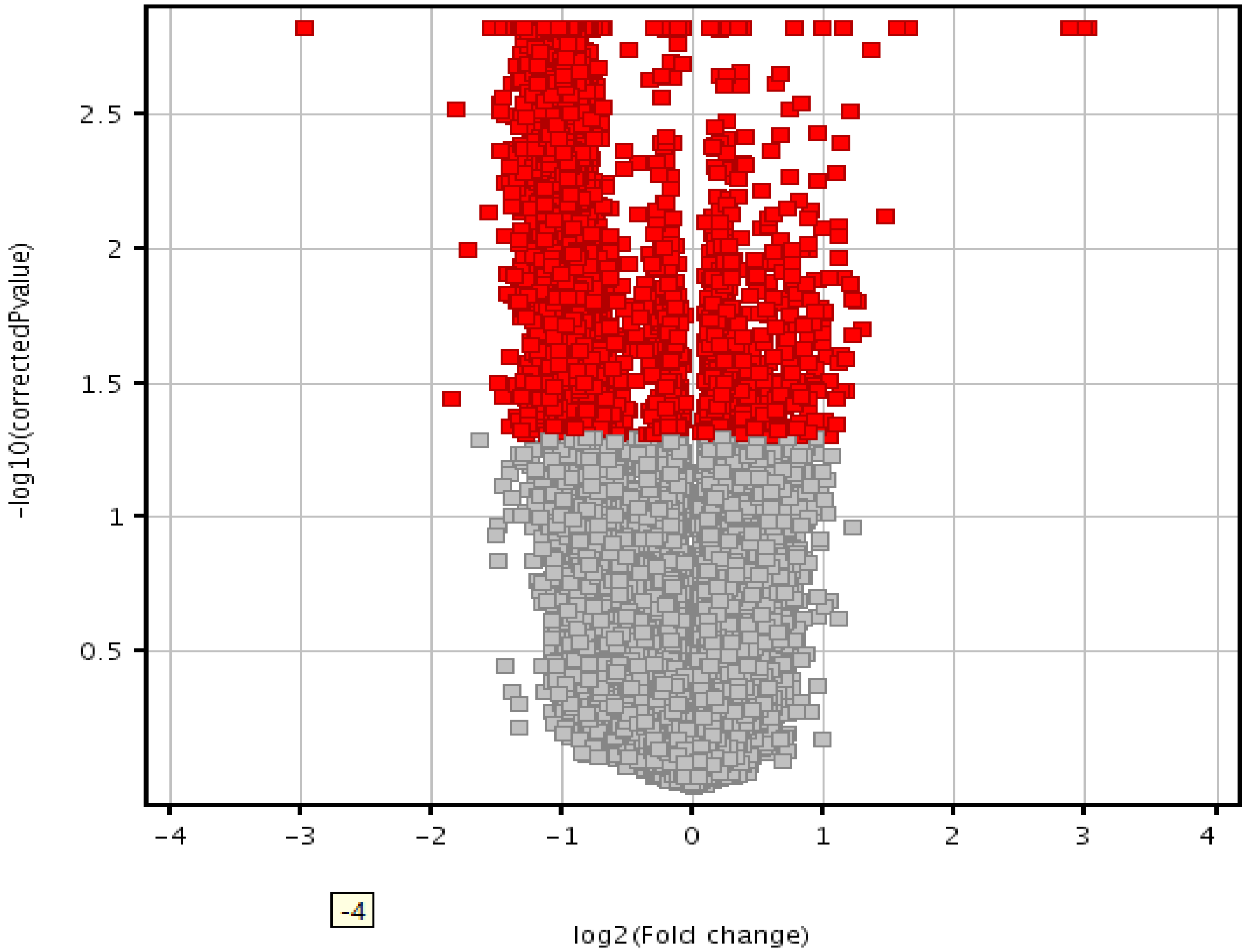

48 h

Test Description

Selected Test : T Test Against Zero
p-value computation: Asymptotic
Multiple Testing Correction: Benjamini-Hochberg

Result Summary

	P all	P < 0.05	P < 0.02	P < 0.01	P < 0.0050	P < 0.0010
FC all	37538	10963	8932	7808	6932	5512
FC > 1.1	20281	10246	8621	7643	6843	5496
FC > 1.5	9676	8202	7418	6830	6304	5285
FC > 2.0	6565	6522	6418	6225	5957	5201
FC > 3.0	49	48	45	43	39	23
Expected by ch...		548	178	78	34	5

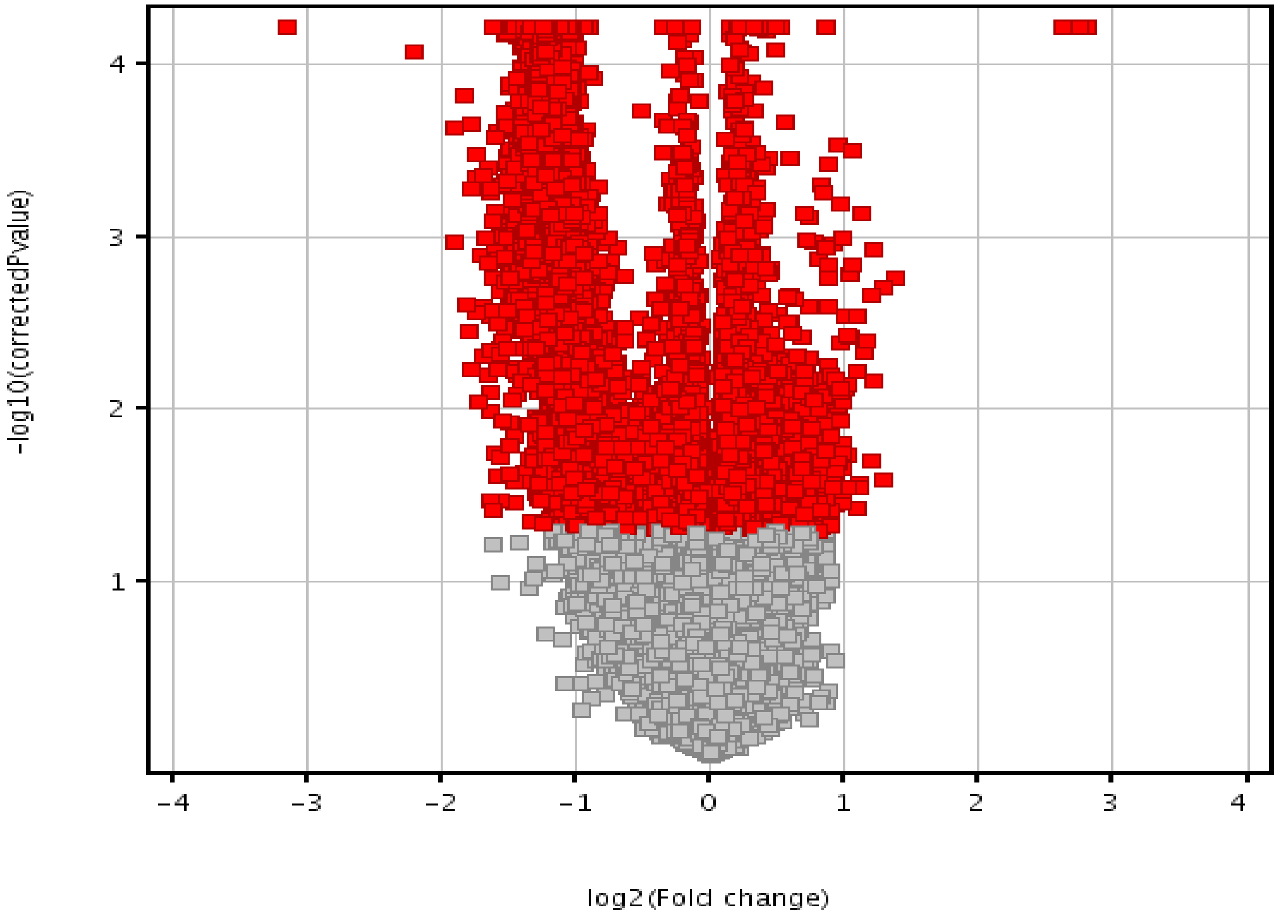

96 h

Test Description

Selected Test : T Test Against Zero
p-value computation: Asymptotic
Multiple Testing Correction: Benjamini-Hochberg

Result Summary

	P all	P < 0.05	P < 0.02	P < 0.01	P < 0.0050	P < 0.0010
FC all	37538	8124	6555	5751	5058	0
FC > 1.1	21772	7853	6446	5699	5037	0
FC > 1.5	9940	6966	6042	5458	4918	0
FC > 2.0	5991	5662	5350	4996	4613	0
FC > 3.0	61	43	34	25	15	0
Expected by ch...		406	131	57	25	0

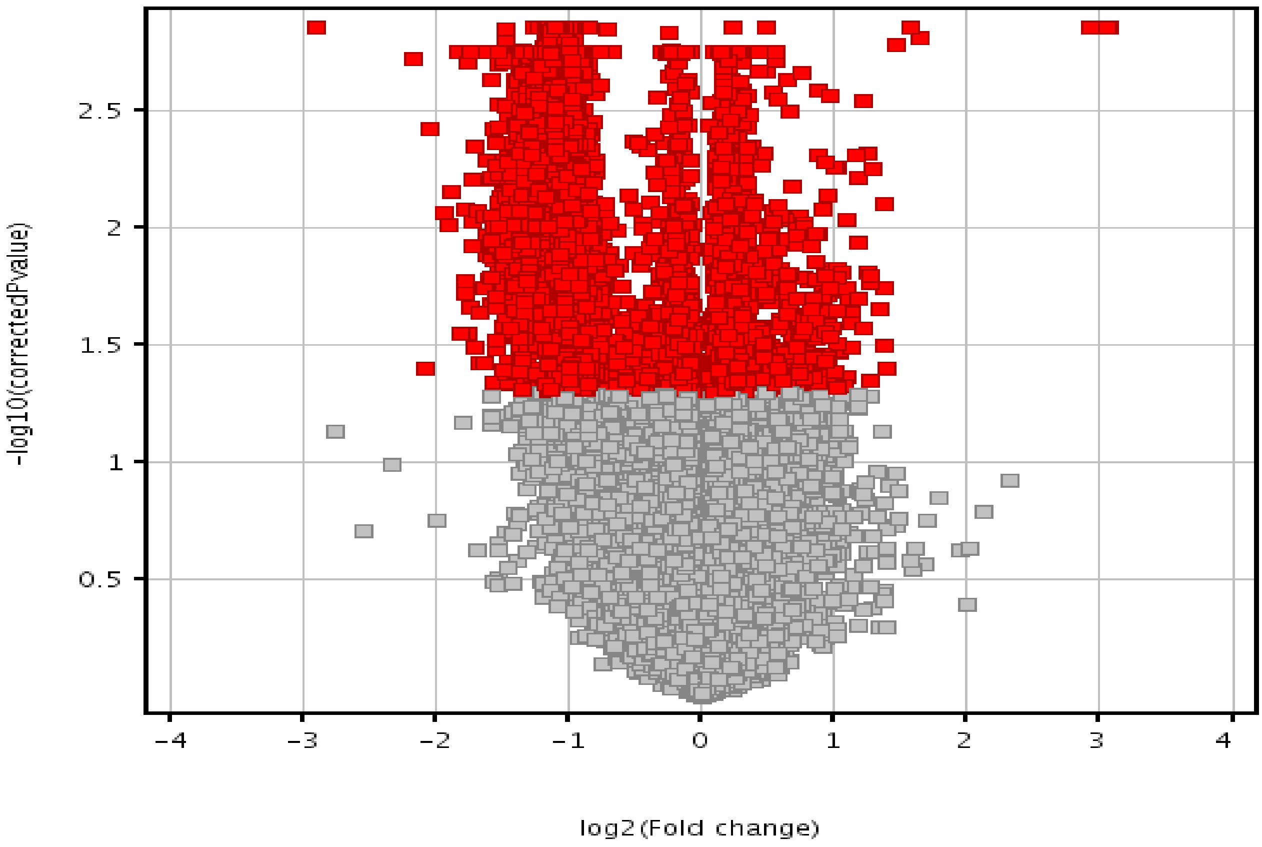

GeneSpring analysis of gene expression from a time course analysis of Arabidopsis thaliana seedlings exposed to GMF (control) and NNMF.

Specification	Time-course point						
	10 min	1 h	2 h	4 h	24 h	48 h	96 h
Number of Biological replicates retained in the analysis (total number)	10 (12)	7 (8)	8 (8)	8 (8)	7 (8)	8 (8)	6 (8)
Number of genes satisfying a corrected P-value cut-off of 0.05 (% out of 37538 genes)	10,811 (29%)	10,321 (27%)	10,876 (29%)	11,135 (30%)	7,984 (21%)	10,963 (29%)	8,124 (22%)
Number of genes with FC > 2 and P < 0.05 (% out of satisfying genes)	3,591 (33%)	6,653 (64%)	6,248 (57%)	2,018 (18%)	4,104 (51%)	6,522 (59%)	5,662 (70%)
Number of genes with FC > 3 and P < 0.05	136	25	19	16	11	48	43
