# Supplementary material for: Transcriptomics and Metabolomics of Reactive Oxygen Species Modulation in Near-Null Magnetic Field-Induced Arabidopsis thaliana
Source: Biomolecules. 2022 Dec 6;12(12):1824. doi: 10.3390/biom12121824 (PMC9775259; doi:10.3390/biom12121824)
Supplement: Supplementary file 1 [file biomolecules-12-01824-s001.zip › Supplementary Table S1.pdf]

Supplementary Table S1: List of primers used for validation using qRT-PCR.

Gene	Locus	Sequence 5′→3′	
ACT1	*At2g37620*	TGCACTTCCACATGCTATCC	Forward
ACT1	*At2g37620*	GAGCTGGTTTTGGCTGTCTC	Reverse
eEF1Balpha2	*At5g19510*	ACTTGTACCAGTTGGTTATGGG	Forward
eEF1Balpha2	*At5g19510*	CTGGATGTACTCGTTGTTAGGC	Reverse
GAPC2	*At1g13440*	TCAGGAACCCTGAGGACATC	Forward
GAPC2	*At1g13440*	CGTTGACACCAACAACGAAC	Reverse
UBP6	*At1g51710*	GAAAGTGGATTACCCGCTG	Forward
UBP6	*At1g51710*	CTCTAAGTTTCTGGCGAGGAG	Reverse
PER14	*At2g18140*	ACGCCTTAACTCTAGCTGCT	Forward
PER14	*At2g18140*	ACAAGTTGTCGGGTTCAGGA	Reverse
GULLO1	*At1g32300*	CGTGCAGCCAAAGTTTCTCT	Forward
GULLO1	*At1g32300*	TTTGCCTTGGCTTGGTAACC	Reverse
MSD2	*At3g56350*	AACCACCGCATGATCCTTTG	Forward
MSD2	*At3g56350*	CTGGTTGGCTGTGGTTTCAA	Reverse
